# A Study on Sexual Dimorphism of Adult Wet Scaphoid Specimens

**DOI:** 10.7759/cureus.22263

**Published:** 2022-02-15

**Authors:** Karthikeyan Gurusamy, Zareena Begum, Yuvaraj Maria Francis, Balaji Karunakaran, Gunapriya Raghunath

**Affiliations:** 1 Anatomy, Saveetha Medical College, Chennai, IND

**Keywords:** sexual dimorphism, scaphoid, morphometry, fracture, avascular necrosis

## Abstract

Background

Sexual dimorphism in human bones has mostly been confined to the study of the bony pelvis. However, considerable changes also exist in the other parts of the skeletal system. This study focuses on the differences in the morphometry of male and female adult scaphoids.

Aims and objectives

We aim to study the morphometry of male and female adult human scaphoids and determine the differences between the same.

Materials and methods

A total of 100 scaphoids were freshly dissected from both hands of 25 male and 25 female cadavers in Saveetha Medical College, Chennai, India. The soft tissues were loosened using 5% potassium hydroxide (KOH) solution. Further, the soft tissues were removed by meticulous dissection. For the measurements, vernier calipers and threads for circumference measurement were used.

Results

The morphometric parameters included length, proximal width, width of the middle part (waist), distal width and circumference of the waist, and circumference of the tubercle in scaphoids. Statistical differences were found in most of the parameters.

Conclusion

A statistically significant difference exists between the morphometric measurements of male and female adult scaphoids, which may prove helpful in the fracture fixation of the scaphoid, as scaphoid fracture has a risk of avascular necrosis.

## Introduction

The human hand is unique in the way that it is dexterous with an opposable thumb. The skeleton in the hand comprises a total of 27 bones, with eight carpal bones in two rows, five metacarpals, and 14 phalanges. The largest of the proximal row carpal bones is the scaphoid, which articulates with trapezium that forms the very important, first carpometacarpal joint, an important articulation for thumb function [1]. The scaphoid presents a long axis that is slightly palmar in direction. It also presents a tubercle that is directed anterolaterally. The dorsal surface of the scaphoid is rough and grooved [2]. The dorsal surface is usually pierced by minute nutrient foramina, which is mostly found in the distal half. The retrograde branches of the radial artery provide the main blood supply for the scaphoid [3]. Since the blood supply to the scaphoid is from distal to proximal, a break in the bone leads to the disruption of blood supply to the proximal pole, which may lead to avascular necrosis [4]. The most common scaphoid fracture encountered is waist fracture, followed by the dorsal sulcus, and finally the proximal pole [5].

In the case of scaphoid fracture, some differences can be found in the dimensions between the male and female scaphoids [6,7]. The data pertaining to sexual differences in the scaphoid is scanty. Therefore, we believe that this study, showing the differences in the dimensions of the scaphoid based on sex, will be helpful for orthopedic surgeons and radiologists in diagnosing and treating pathologies of the scaphoid, wherein surgical fixation and implants can be custom decided according to the gender-specific dimensions. Radiologic studies on the morphometry of the scaphoid, irrespective of gender, are available [8]. Dry bone studies cannot ascertain sexual dimorphism in scaphoids; thereby, freshly dissected scaphoid specimens were taken for this study.

## Materials and methods

Study duration, sample size, and sampling technique and method

After obtaining ethical approval from the Institutional Ethical Review Board (IERB) of Saveetha Medical College (approval number: SMC/IEC/2021/03/065) dated February 20, 2021, this study was conducted in February 2021. A total of 100 freshly dissected scaphoid specimens from both sides of 25 male and 25 female cadavers were taken for this study. Non-probability convenience sampling was employed in the analysis.

**Figure 1:**
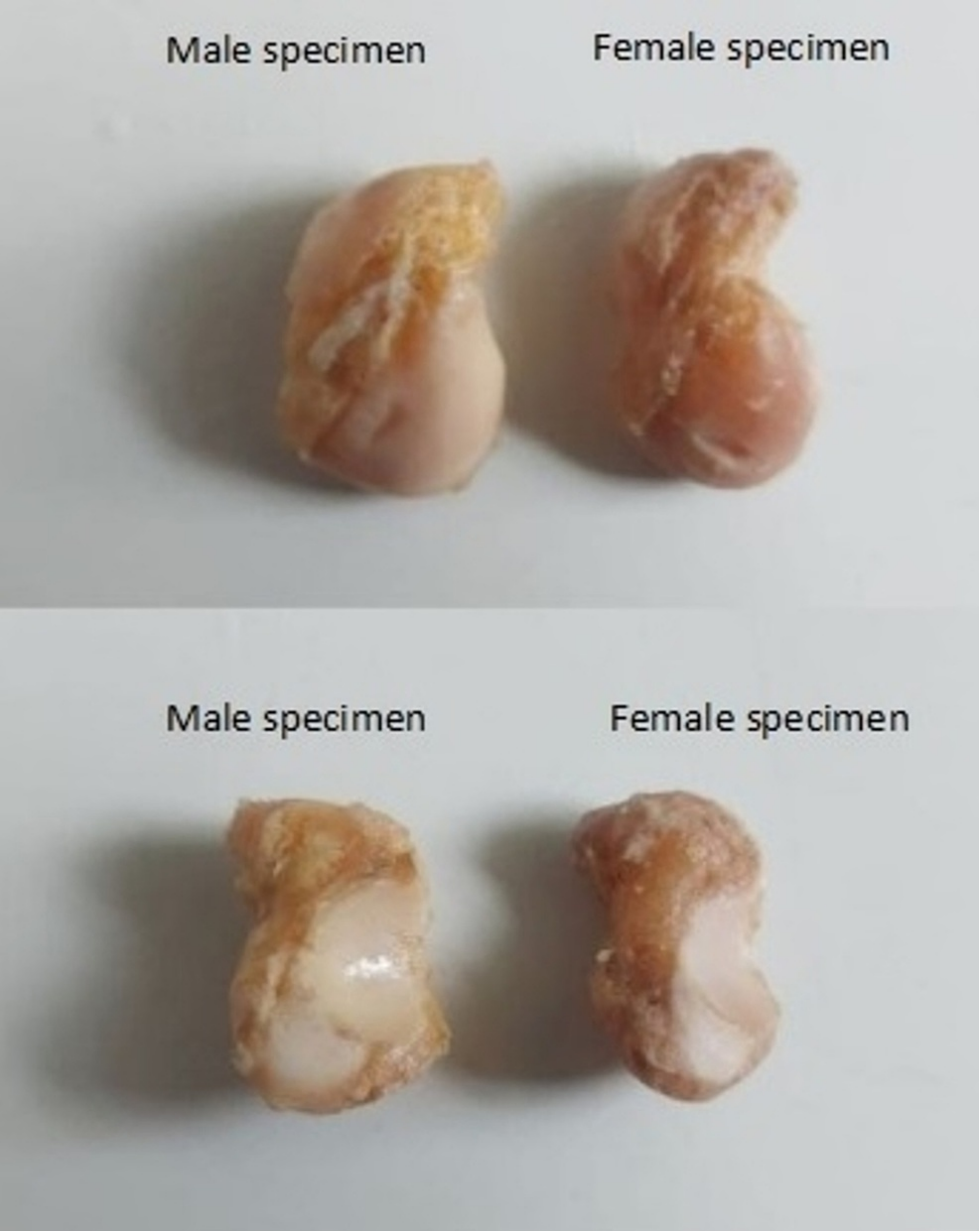
Dissected male and female scaphoid specimens with soft tissues cleared out. The images above and below show the dorsal aspect and the palmar aspect, respectively, of the scaphoid specimens in both sexes.

The dissection was started with the palpation of the scaphoid in the lateral aspect of the radial lower end, followed by the removal of the proximal part of the thumb. The extensor tendons were retracted, and after excising all the ligamentous connections, the scaphoids were carefully removed, further followed by the dissection of the soft tissues of the scaphoids. For the complete removal of the soft tissues from the bone, 5% potassium hydroxide (KOH) solution was used, and the scaphoids were immersed in the same for five minutes. Then, running water and scrubbing rendered the scaphoids free of soft tissues, and the scaphoids were then dried as shown in Figure 1.

Data collection and data analysis

The morphometric parameters were entered in a Microsoft Excel spreadsheet (Microsoft® Corporation, Redmond, WA, USA) for male and female specimens. The instruments used include vernier calipers and a non-expandable and dry thread for measuring the circumference. The thread is marked with points using markers when the circumference is measured and then straightened, and the distance between the points was measured using a scale.

Morphometric Measurements

**Figure 2:**
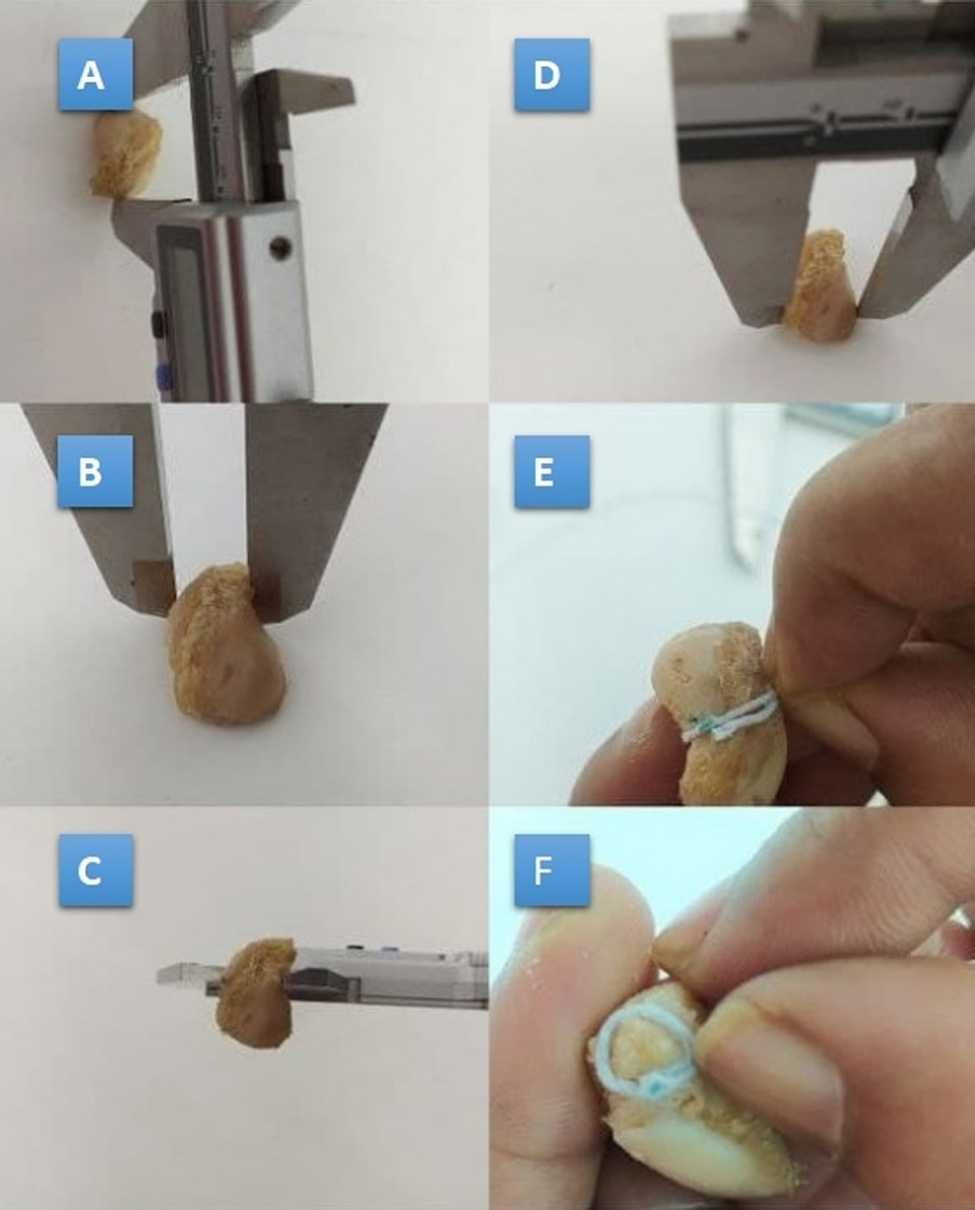
Morphometric analysis of male scaphoid specimens. A: Measurement of the length. B: Measurement of the proximal width. C: Measurement of the waist. D: Measurement of the distal width. E: Measurement of the circumference of the waist. F: Measurement of the circumference of the tubercle.

**Figure 3:**
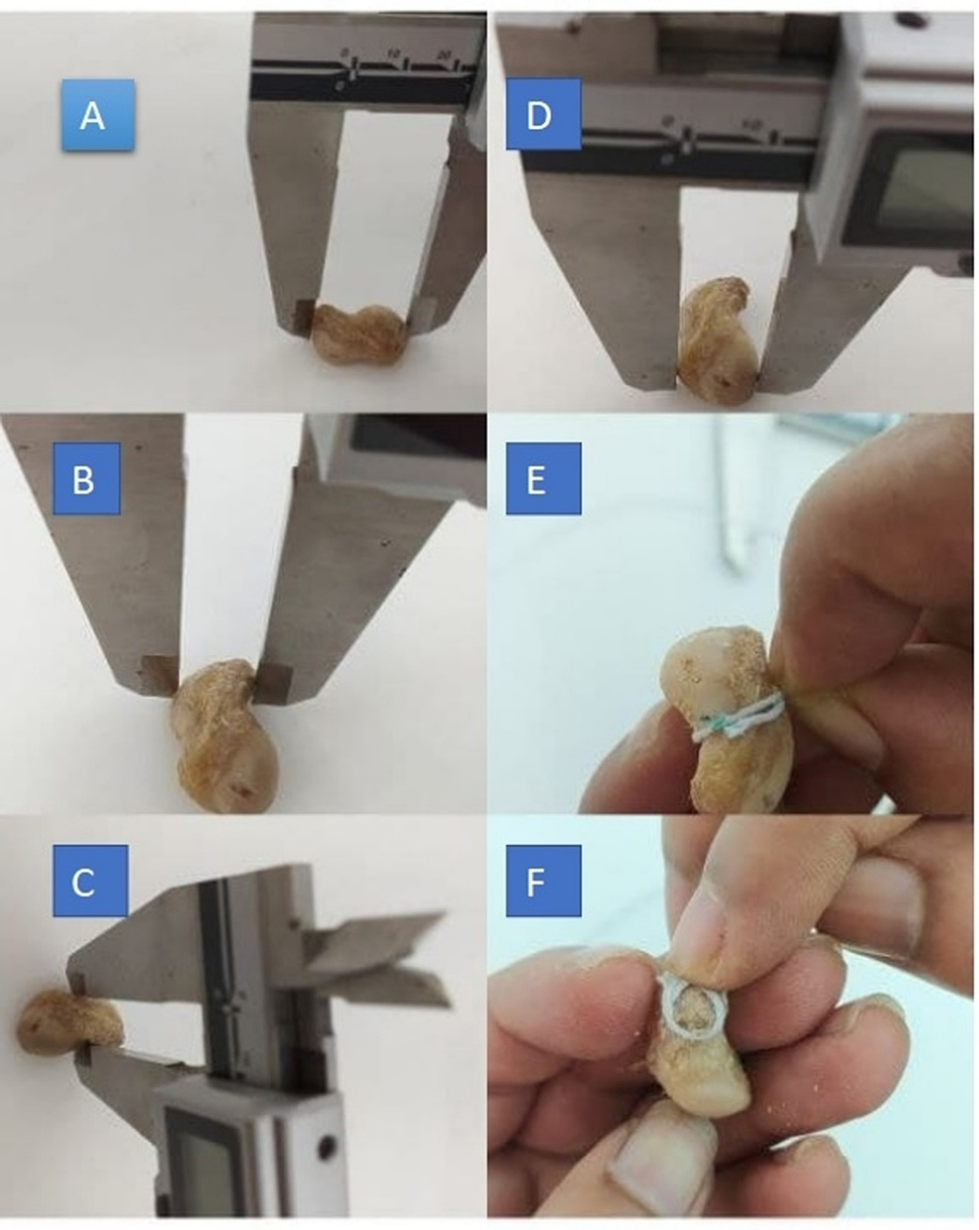
Morphometric analysis of female scaphoid specimens. A: Measurement of the length. B: Measurement of the proximal width. C: Measurement of the waist. D: Measurement of the distal width. E: Measurement of the circumference of the waist. F: Measurement of the circumference of the tubercle.

The length was calculated between the prominent points in the proximal surface and the prominent part of the tubercle. The width was measured at three levels: proximal width (maximum width at the proximal level), waist (width of the narrowest part), and distal width (width of the widest part of the distal part). The circumference of the waist (narrowest point) and the circumference of the tubercle (base level of the tubercle) were measured using a thread and a scale. All measurements were taken in both male and female scaphoid specimens as shown in Figure 2 and Figure 3.

Data were collected, tabulated, and analyzed using descriptive statistics and Student’s t-test using Microsoft Excel 2013. A P-value of less than 0.05 was taken as significant.

## Results

Length of the scaphoids

The mean length of the scaphoids of the male specimens was found to be 27.55 mm with a standard deviation of 1.323, and the mean length of the scaphoids of the female specimens was 24.47 mm with a standard deviation of 1.313. The P-value derived using t-test showed significance as shown in Table 1.

Width of the scaphoids

Proximal Width

The mean proximal width of the scaphoids of the male specimens was found to be 27.55 mm with a standard deviation of 1.323, and the mean proximal width of the scaphoids of the female specimens was 24.47 mm with a standard deviation of 1.313. The P-value derived using t-test showed significance as shown in Table 1.

Width of the Waist

The mean waist width of the scaphoids of the male specimens was found to be 9.12 mm with a standard deviation of 0.571, and the mean waist width of the scaphoids of the female specimens was 8.45 mm with a standard deviation of 0.693, which were statistically significant as shown in Table 1.

Distal Width

The mean distal width of the scaphoids of the male specimens was found to be 10.32 mm with a standard deviation of 0.752, and the mean distal width of the scaphoids of the female specimens was 9.39 mm with a standard deviation of 0.364, which were statistically not significant as shown in Table 1.

Circumference of the waist

The mean circumference of the waist of the male specimens was found to be 3.8 cm with a standard deviation of 0.229, and the mean circumference of the waist of the female specimens is 3.37 cm with a standard deviation of 0.204, which showed statistical significance as shown in Table 1.

Circumference of the tubercle

The mean circumference of the tubercle of the male specimens is found to be 2.56 cm with a standard deviation of 0.223, and the mean circumference of the tubercle of the female specimens is 2.3 cm with a standard deviation of 0.104. The P-value derived using t-test showed no significance as shown in Table 1.

**Table 1:** Mean values of the morphometric analysis of the male and female scaphoids, with their significance.

	Male (in mm) (mean±SD)	Female (in mm) (mean±SD)	P-value
Length	27.55±1.323	24.47±1.313	0.00149 (highly significant)
Proximal width	12.86±1.0800	11.52±0.795	0.0086 (significant)
Waist width	9.12±0.571	8.45±0.673	0.0272 (significant)
Distal width	10.32±0.752	9.39±0.364	0.0749
Circumference of the waist	38.0±0.229	33.7±0.204	0.0089 (significant)
Circumference of the tubercle	25.6±0.223	23.0±0.104	0.0786

## Discussion

Overall, scaphoid fractures account for 2%-7% of all types of fractures encountered. Scaphoid fractures are the commonest at 60%-70% among the carpal bones to get fractured [9]. Misdiagnosis of scaphoid fractures is common, where they can be diagnosed as a wrist strain. Chances of nonunion increase in the case of displaced fracture. Misdiagnosis can lead to nonunion in the case of fracture that is displaced, and further, it may lead to arthritis, instability of the wrist, and deformity. Scaphoid injuries occur predominantly in the young adult age group, with an average age of 29 years [10].

On the treatment front of scaphoid fractures, nondisplaced fractures and fractures that are confined to the distal third of the bone can be treated with conservative management [11]. Conservative management involves the use of a long or short arm cast with a thumb spica to immobilize the thumb [12]. Operative treatment of scaphoid includes some indications, a few of which include displacement of fracture segment of more than 1 mm, humpback deformity (where the intra-scaphoid angle is more than 35°), proximal pole fracture, comminuted fractures, fractures showing nonunion, and avascular necrosis. In this procedure, the crucial step involves the positioning of the screw, which should be in the middle third of the central axis of the scaphoid, which improves union and alignment and also provides the most stability.

The surgical approach for scaphoid fractures involves the use of single or multiple screws, which can be done percutaneously in the case of fracture with nil or minimal displacement or through an open procedure in the case of nonunions or in fractures with more displacement [13]. The prognosis for a fracture with less or nil displacement holds a union rate of 90% [14].

Sexual dimorphism of scaphoids in wet specimens has not been sufficiently documented. For the intraoperative fixation of scaphoid fractures, the scaphoid is explored by the volar or dorsal approach, which is predominantly dependent on the location of the fracture and its type [15,16]. Both approaches have shown complications [17,18]. The screw that is selected for internal fixation should have a length where it can be placed 2 mm under the surface, both at proximal and distal poles, which is a clinically relevant point to avoid chondral wear in the bone [19]. Thereby, it becomes essential to customize the implant as per the size of the scaphoid. Significant differences in the length of the scaphoids between male and female scaphoid specimens, as shown in Table 2, were found in the studies conducted by Heinzelmann et al. (2007) [7], Kigera et al. (2017) [20], and Meermans et al. (2012) [21].

**Table 2:** showing the comparison of length of male and female specimens of scaphoids with previous studies. Lengths are measured in mm.

	Present study	Meermans et al. (2012) [21]	Heinzelmann et al. (2007) [7]	Kigera et al. (2017) [20]
Mean length in male specimens	27.55	27.14	31.3	32.7
Mean length in female specimens	24.47	23.86	27.3	27.3

All the dimensions of scaphoids, as measured in the current study, were compared with a previous study done by Heinzelmann et al. (2007) as shown in Table 3. The morphometric variations of scaphoids were first reported in the study of Ceri et al. (2004), where the average length was found to be 25.8 mm and the waist width was found to be 10.8 mm [22], whereas in an Indian study done by Purushothama et al. (2011) [23] and Babu (2019) [24] showed the average length of scaphoids to be 22.65 and 24.65 mm, respectively, both of which were dry bone studies where the gender of the specimen was unknown.

**Table 3:** Comparison of the dimensions of scaphoids. The measurements of all the dimensions of scaphoids of the current study and those of Heinzelmann et al. (2007) were compared. All dimensions are measured in mm.

	Present study	Heinzelmann et al. (2007) [7]
Mean length (male)	27.55	31.3
Mean length (female)	24.47	27.3
Mean proximal pole width (male)	12.86	4.5
Mean proximal pole width (female)	11.52	3.7
Mean distal pole width (male)	10.32	7.2
Mean distal pole width (female)	9.39	7.2
Mean waist width (male)	9.12	13.6
Mean waist width (female)	8.45	11.1

This study brings out the sexual dimorphism of scaphoids in wet specimens rather than dry specimens, where the availability of data is less; this study also has a sufficient sample size. The limitations of the study can be a concomitant 3D analysis using radiographic techniques so that the screw placement plane can be correlated to morphometry. This study involves the specimens of the South Indian population; therefore, the scope of the study can be increased by further studies.

## Conclusions

The present study showed a statistically significant difference in the length, proximal width, and waist of scaphoids. This study is specific to the South Indian population; therefore, further studies in other regions can be collated for further analysis and data. These differences show that internal fixation using the same implant in both males and females can lead to a longer implant in a smaller bone. Thereby, the current study brings out an analysis of the differences in the morphometry of male and female scaphoid specimens, which can be helpful to radiologists and treating orthopedic surgeons in customizing the implant for effective union post fracture without complications.
